# Benefits for Nurses

**Published:** 1913-03-08

**Authors:** 


### NATIONAL INSURANCE ACT DEPARTMENT.

### XLV.—Benefits for Nurses.

THE announcement which we were able to make last week that the Insurance Commissioners had, through the Insurance Committees, allowed hospital employees to make their own arrangements in regard to medical benefit was distinctly gratifying, as showing that the special claims of institutional workers are receiving attention at the hands of the Insurance authorities. These claims to special consideration have been consistently advocated in our columns, particularly in regard to the nursing staffs of the hospitals, and we are glad to know that the cause has at last received some recognition. The effect of this decision is that the nurses concerned will not be required to place their names on the lists of patients of panel doctors, and they will be at liberty to make their own arrangements with members of the hospital medical staffs.

It has to be admitted, however, that even under this arrangement the nurses have to pay for treatment whilst ill that they formerly obtained gratuitously in part payment for their services.

It may be recalled that in our issue of the previous week we made the suggestion that the most satisfactory solution to this difficult matter would be by allowing the nurses to contract out of medical benefit whilst employed in hospitals. We still hold to this view, and feel strongly that an effort should be made to incorporate a clause to this effect in the first amending Act that may be brought forward. We indicated that by contracting out of medical benefit the contribution could be reduced by 1½d. a week. This means, of course, that the combined contribution of the employer and employee would be reduced by that amount. The figure is arrived at quite simply, because the weekly contribution payable in England, Scotland, and Wales is 1½d. more than that payable in Ireland, and the sole difference in the benefits is that medical treatment and attendance is provided in the former case but not in the latter.

In the case of nurses we hold that the full advantage of the reduction in contributions should be given to them, and not shared between the hospital authorities and the nurses. Thus, the advantage given to the nurses under the arrangement we suggest would be the reduction of their contributions by one-half whilst they are in hospital service.

### EXCLUSION OF NURSES FROM INSURANCE.

Mention was made of the special treatment accorded to elementary school teachers under the Act, and it was claimed that if their requirements demanded special consideration there was no reason why the peculiar circumstances of hospital nurses should not also obtain attention. In the case of the elementary school teachers the special treatment was to exclude them from the compulsory provisions of the Act. This was done on the ground that they were already required to make compulsory contributions to a deferred annuity fund; and, further, that, in a sense, they were already provided for in the event of disablement through the disablement annuity which may be claimed from the Government under certain conditions. These conditions do not apply to nurses, while it is suggested that they should be entirely excepted from compulsory insurance under the Act. One point that must be borne in mind in this connection is that, although there may be no necessity for sickness insurance whilst a nurse remains in hospital service, there is no like provision for her when she leaves that service except through the Royal National Pension Fund. It is then that the advantage of the insurance might be felt, for when she is ill a nurse is, from the nature of her work, debarred from earning her usual means of livelihood, and the sickness and disablement benefits might in such circumstances prove of value. A further point that has to be considered is that sickness tables show conclusively that sickness rates increase with age, and in consequence of this the small weekly contributions paid, with assistance from employers, in early life provide the considerable sums necessary to meet the claims for benefit in advancing years.

### THE NURSES' SOCIETY.

With regard to sickness benefit for nurses—that is to say, the payment in money by the approved societies whilst the nurses are rendered incapable of work by illness or disablement—there is every reason to believe that, if our recommendations have been followed in regard to the choice of a society, substantial advantages will ultimately accrue. The Nurses' Insurance Society, like all other approved societies, has had to commence its operations by granting sickness benefit at the rate mentioned in the Act—namely, 7s. 6d. a week in normal cases. By its rules this Society is confined exclusively to female nurses with hospital training, and it is a reasonable inference that the members will be a particularly "select" body of lives—using the term "select" in its technical sense as meaning a class of lives subject to specially favourable rates of sickness. Then, again, it follows as a necessary consequence of the constitution of the Society that claims for maternity benefit will be small. Thus there will be at least two important sources from which profits are likely to accrue, and as all profits must be distributed wholly among the members in the form of additional or increased benefits, there is every prospect that the Nurses' Insurance Society will be in a particularly favourable position when the first valuation is made, and that the benefits will progressively increase for some years to come. We understand that it has been decided that, although nurses employed in hospitals receive treatment when ill in the hospital buildings, they are not debarred on that account from the receipt of sickness benefit from their approved societies.

### New Arrangements in Hospitals.

Owing to the frequent changes in the medical staff in hospitals, a difficulty might arise as to the form of agreement between the Insurance Committee and the practitioners concerned. It was, therefore, suggested by the Nurses' Insurance Society that the agreement might be made between the Committee and the "resident medical officer for the time being" of the hospital, and not with the doctor by name. The Insurance Commissioners have accordingly agreed to the 'resident medical officer of a hospital or similar institution acting in respect of that institution for insured persons, and that for this purpose the title of the office alone will be inserted in the panel, with an asterisk indicating that the medical officer is only available for persons on the staff of the institution. The London Insurance Committee have further intimated that it is proposed to provide special forms of agreement in such cases owing to the fact that there will be frequent changes in the medical staff. This arrangement will enable the hospital authorities to secure the capitation allowance for medical treatment by an arrangement with their "medical officer." This arrangement will equally apply as regards the dispensing of drugs, etc., wherever a qualified chemist is employed by the institution. In this case it will also be possible for the "office of the chemist for the time being" to be added to the panel, with an asterisk indicating that the supply of drugs is limited only to the staff of the hospital. In many hospitals the nursing staff is attended during sickness by someone not on the resident medical staff; even in such cases the above arrangement will still hold good, and doctors who have hitherto attended the staff can treat them just as they have done in the past; but, in order that the hospital may secure the capitation allowance, the formal documents required by the Committee would have to be signed by the "resident medical officer" on the limited panel. As this arrangement has the sanction of the Commissioners, probably all Insurance Committees will see their way to make this consideration for the benefit of hospitals.

### Inmates of Charitable Institutions.

Whilst dealing with the question of institutional treatment it may be interesting if we briefly refer to the provisions of Section 51. This section deals with the insurance of persons who are inmates of institutions carried on for charitable or reformatory purposes, and provides that when such persons receive maintenance and medical attendance when sick, the Insurance Commissioners may grant a certificate of exemption to the managers excepting such persons from insurance. In such circumstances no contributions will be payable in respect of the inmates as insured persons either by the managers of the institutions or the inmates themselves. Before a certificate of this description is given the Insurance Commissioners have to be satisfied that due provision is made for maintenance and medical treatment, and further conditions are laid down, in somewhat ambiguous terms, the purport of which appears to be that the managers must undertake the liability of having to pay in respect of every person who has been an inmate for more than six months such capital sum as will enable him to enter or re-enter insurance, on leaving the institution, at full benefit rates without any disqualification on account of the contributions not paid while he was an inmate.

The advantages to be obtained under this section depend on the circumstances of the institution concerned, and it is not easy to give a general statement of the benefits that will be derived by the managers in obtaining the certificate of exemption.

Whilst the person is an inmate the institution is saved from the necessity of paying the contributions, but if the inmates as a general rule are members of approved societies before entering the institutions, and stay on the average for more than six months, this advantage will most likely be more than counterbalanced by the capital payments that have to be made on leaving.

It has further to be borne in mind that the capital payments not only refer to the employer's part of the contributions, but also to that of the employee. In such circumstances it would be impossible to obtain reimbursement for the employee's part of the contribution, and thus there may be conditions under which the adoption of the provisions of the section would not only result in the institution receiving no gain, but they might actually suffer loss thereby. There are, doubtless, many institutions which will be benefited by obtaining certificates of exemption. As an illustration, we might instance the case of charitable industrial homes for the blind and deaf and dumb, where the inmates frequently obtain permanent employment, and for whom it has been customary to provide maintenance and medical treatment when ill.

**NOTE.—Questions and Correspondence on all doubtful points are invited, and should be addressed to the Editor, The Hospital, 28, 29 Southampton Street, Strand, London, W.C., and marked "Insurance."**

